# Pattern of failure and clinical value of local therapy for oligo‐recurrence in locally advanced non‐small cell lung cancer after definitive chemoradiation: Impact of driver mutation status

**DOI:** 10.1002/cam4.5493

**Published:** 2022-12-16

**Authors:** Jinmeng Zhang, Jiuang Mao, Dayu Xu, Shanshan Jiang, Tiantian Guo, Yue Zhou, Li Chu, Xi Yang, Xiao Chu, Jianjiao Ni, Zhengfei Zhu

**Affiliations:** ^1^ Department of Radiation Oncology Fudan University Shanghai Cancer Center Shanghai China; ^2^ Department of Oncology, Shanghai Medical College Fudan University Shanghai China; ^3^ Institute of Thoracic Oncology Fudan University Shanghai China; ^4^ Shanghai Clinical Research Center for Radiation Oncology Shanghai China; ^5^ Shanghai Key Laboratory of Radiation Oncology Shanghai China

**Keywords:** definitive chemoradiation, driver mutation, local therapy, non‐small cell lung cancer, oligo‐recurrence

## Abstract

**Introduction:**

Considerable differences of treatment response and pattern of failure may exist between definitive chemoradiation (CRT) treated locally advanced non‐small cell lung cancer (LA‐NSCLC) patients. The clinical value of additional tyrosine kinase inhibitors (TKIs) before disease recurrence and salvage local therapy after initial recurrent disease remain controversial.

**Methods and Materials:**

Consecutive LA‐NSCLC patients receiving definitive CRT and having definite results about driver mutations (EGFR, ALK and ROS1) were retrospectively reviewed. Initial recurrent disease was classified as in‐field recurrence, out‐of‐field recurrence and distant metastasis. Recurrent disease occurred only in the brain or limited to ≤3 extra‐cranial organs and ≤5 extra‐cranial lesions, was defined as oligo‐recurrence. Progression free survival and overall survival (OS) were calculated from diagnosis to disease progression or death, and to death, respectively. OS2 was measured from initial disease recurrence to death among patients who had recurrent disease.

**Results:**

Of the 153 enrolled patients, 39 had driver mutations and 13 received additional TKI therapy besides definitive CRT. Patients harboring driver mutations but without additional TKI therapy had a similar PFS and significantly longer OS (*p* = 0.032) than those without driver mutations. Additional TKI therapy prolonged PFS (*p* = 0.021) but not OS among patients with driver mutations. No significant difference of pattern of failure was observed between patient subgroups stratified by the status of driver mutations and the usage of additional TKI therapy. Furthermore, 57 of the 95 patients with initial recurrent disease developed oligo‐recurrence and salvage local therapy significantly improved OS2 (*p* = 0.01) among patients with oligo‐recurrence disease.

**Conclusion:**

LA‐NSCLC patients receiving definitive CRT generally had similar PFS and pattern of treatment failure, regardless of driver mutation status. Additional TKI therapy besides definitive CRT could prolong PFS but not OS. The majority of recurrent disease after definitive CRT belongs to oligo‐recurrence and salvage local therapy may provide survival benefit.

## Graphical abstract

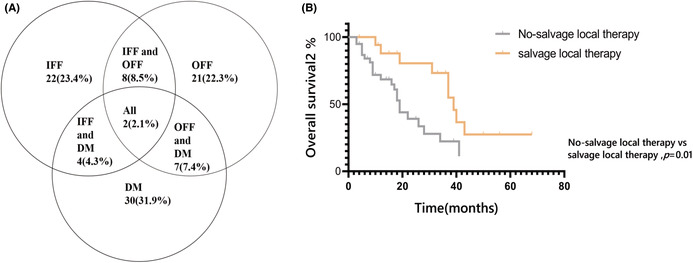

## 1 INTRODUCTION

Based on the encouraging survival results from the PACIFIC trial, definitive concurrent chemoradiation (CRT) plus durvalumab consolidation have become the new standard of care for inoperable locally advanced non‐small cell lung cancer (LA‐NSCLC).^1^, ^2^ However, various studies found that patients with driver mutations, particularly epidermal growth factor receptor (EGFR) mutations, may not benefit from durvalumab consolidation.^3^, ^4^ Considerable differences in treatment response and pattern of failure may exist between definitive CRT‐treated patients with LA‐NSCLC with or without driver mutations.^5^, ^6^, ^7^, ^8^, ^9^, ^10^, ^11^ In several studies, patients with EGFR‐mutant LA‐NSCLC were found to have shorter progression‐free survival (PFS) than those without EGFR mutations,^5^, ^6^, ^7^ whereas another study showed that patients with LA‐NSCLC with EGFR‐sensitizing mutations had a trend toward improvement in the overall response rate (ORR) compared with patients without EGFR mutations, with no significant differences in relapse‐free survival (RFS) and overall survival (OS).^12^ Most patients with inoperable stage III NSCLC would face loco‐regional and/or distant recurrences in the first 2 years following definitive CRT,^2^, ^13^, ^14^, ^15^ with the feasibility and clinical value of salvage local therapy remaining controversial. Metastasectomy or cranial radiation may provide clinical benefit for patients with isolated brain metastases (BMs) following definitive CRT,^16^, ^17^ whereas re‐irradiations are occasionally performed for patients with loco‐regional recurrence.^18^, ^19^, ^20^ However, investigations about the clinical value of local therapy in patients with limited extra‐pulmonary metastatic lesions outside the central nervous system, with or without local recurrence in the thorax, are scarce. Conversely, for patients with NSCLC with oligo‐metastatic disease, local interventions, including surgery, radiotherapy, and radiofrequency, are found to prolong PFS or even OS.^21^, ^22^, ^23^ Based on these findings, we hypothesize that appropriate local therapy may also provide extra survival benefits in patients with oligo‐recurrence, defined as BMs only without extra‐cranial recurrence (i.e., isolated BMs) or limited extra‐cranial recurrent disease without BMs. However, the incidence of patients with oligo‐recurrence following definitive CRT and the survival benefit of salvage local therapy in these patients are rarely reported.

Tyrosine kinase inhibitors (TKIs) targeting specific driver mutations, including EGFR, ALK, and ROS‐1, are widely used for patients with advanced NSCLC harboring corresponding driver mutations, with manageable toxicities and promising treatment efficacies.^24^ However, whether TKIs should be used for patients with LA‐NSCLC remains controversial. To further clarify the clinical value of EGFR–TKIs in this disease population, real‐world data are of great significance as phase 3 clinical trials are highly difficult to accomplish.^25^ This study aimed to explore the impact of driver mutation status on the survival outcomes and treatment failure patterns among patients with LA‐NSCLC receiving definite CRT. Furthermore, the feasibility and clinical value of salvage local therapy among patients with oligo‐recurrence were examined.

## 2 PATIENTS AND METHODS

### 2.1 Patients and treatments

Patients with pathologically confirmed NSCLC, determined to be inoperable with locally advanced NSCLC generally through multidisciplinary discussions, treated with definitive CRT, and had their tumor tissue tested for the status of common driver genes (including EGFR, ALK, and ROS1) at the Department of Pathology of the Fudan University Shanghai Cancer Center from January 2013 to March 2021 were retrospectively enrolled. The specific implementation process of radiotherapy plan, dose requirements for treatment planning, and radiation techniques are described elsewhere.^26^ Definitive CRT was defined as two or more cycles of platinum‐based chemotherapy concurrently or sequentially with curative thoracic radiotherapy (54–66 Gy), wherein the mean dose to the lung was <20 Gy, V20 (the volume of the lung parenchyma that received 20 Gy or more) was <35%, or both. Patients receiving corresponding TKIs in addition to definitive CRT, before disease recurrence, were allowed and those receiving durvalumab consolidation were excluded. The following data were collected from the medical records for each patient at the time of disease diagnosis: age, sex, smoking status, Eastern Cooperative Oncology Group (ECOG) performance status, clinical stage, histology, and the status of pretreatment positron emission tomography–computed tomography (PET–CT). Patients were divided into three groups according to the status of driver mutations and the use of TKI therapy. Patients without driver mutations were included in the “wild‐type group” and those harboring driver mutations and receiving additional TKI therapy were included in the “TKI group,” whereas those with driver mutations although did not receive additional TKI therapy were classified as the “non‐TKI group.”

### 2.2 Follow‐up and pattern of failure analyses

Patients were generally clinically and radiographically followed up every 3 months during the first year and at 6‐ to 12‐month intervals thereafter, with chest CT and ultrasonography of abdominal and cervical regions. Brain magnetic resonance imaging and bone scanning were performed after suggestive clinical signs and symptoms. PET–CT was not mandatory. Treatment responses were assessed using the Response Evaluation Criteria for Solid Tumors, version 1.1.^27^ The morphological changes on CT scan were mainly used in the response assessment, and visual FDG (fluorodeoxyglucose) uptake changes (decrease, stable, and increase) were additionally considered, if available.

The patterns of initial disease progression after definitive CRT were classified into three types based on the location and relationship with the radiation field.^28^ In‐field failure was defined as disease progression of irradiated residual tumors or newly developed radiographic lesions if >80% of the volume was covered by the 60% isodose line of the initial RT(radio therapy) plan (the mean dose received >95% of prescription). New lesions developed in the thoracic region that could not be classified as in‐field failure were defined as out‐of‐field failure (the mean dose received <50% of prescription). Extra‐pulmonary disease spread, including pleural metastasis, was classified as distant metastasis. Oligo‐recurrent disease (ORD) was defined as either only BMs without extra‐cranial recurrence (e.g., isolated BMs) or only extra‐cranial recurrent disease (without BMs) limited to no more than three organs and no more than five lesions.

### 2.3 Statistical analysis

PFS was defined as the time interval from diagnosis to initial disease progression or death due to any cause. OS was defined as the time interval from diagnosis to death due to any cause. Post‐progression survival (OS2) was defined as the time interval from the documentation of initial disease recurrence to death of any cause. The characteristics of patients in the two groups were descriptively compared using chi‐squared test or Fisher's exact tests and *t*‐test or the Mann–Whitney test for categorical and continuous variables, respectively. The survival functions were computed using Kaplan–Meier estimates, and the log‐rank test was employed to analyze survival differences. The correlations of common clinicopathological parameters, including sex, age, ECOG performance status, smoking status, TNM stage, histology, driver mutation status, baseline PET–CT, and salvage local treatment, with the survival end points (including PFS and OS), were analyzed using the univariate Cox proportional hazard regression. Subsequently, parameters with a *p* ≤ 0.1 were included in the multivariate Cox proportional hazard regression analyses. Statistical analyses were performed using Statistical Package for the Social Sciences (SPSS) version 24.0 (SPSS). All tests were bilateral, and *p* < 0.05 was considered statistically significant.

## 3 RESULTS

### 3.1 Patients' characteristics

**FIGURE 1:**
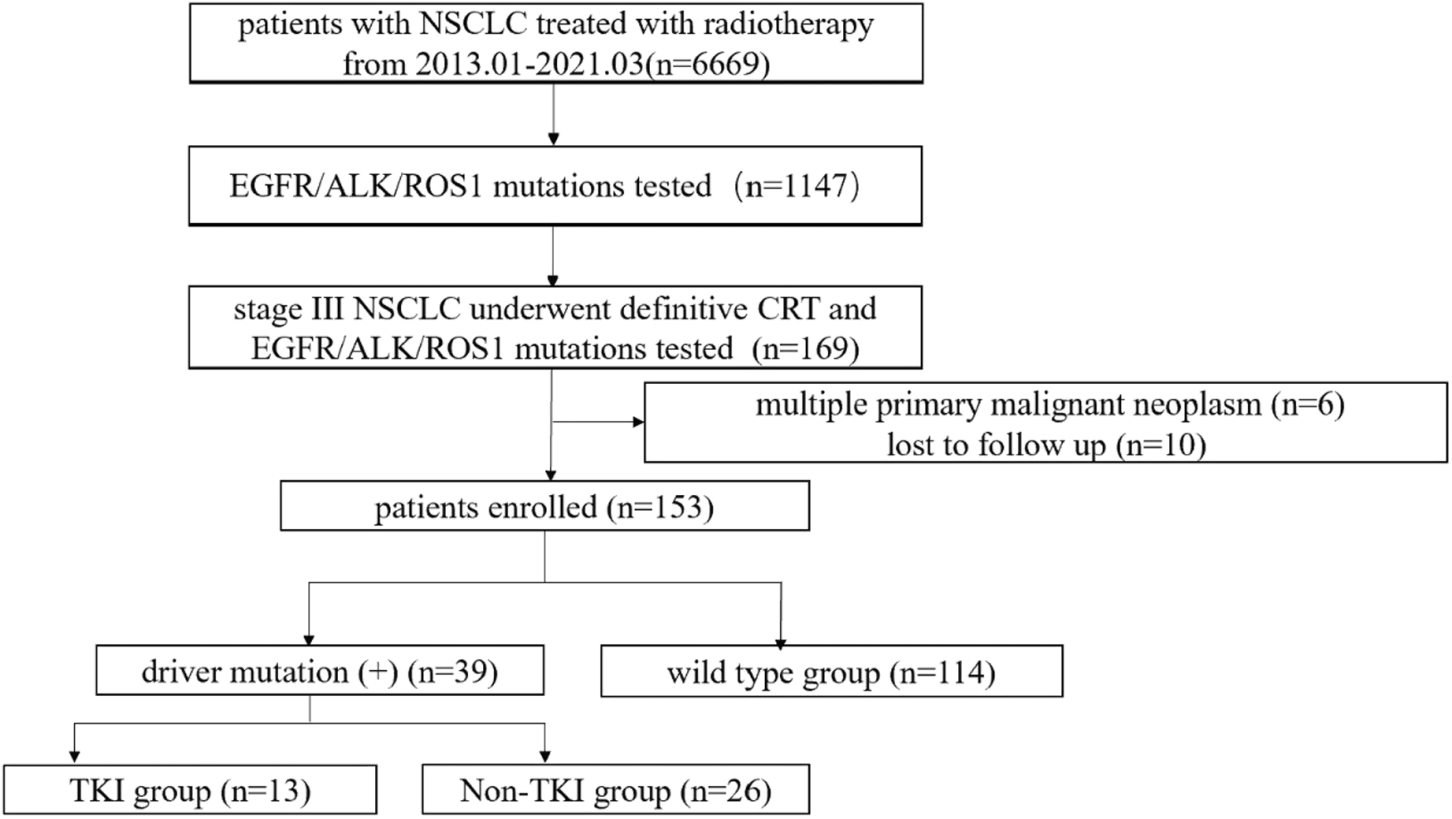
Flow chart of patient enrollment

A total of 153 patients were enrolled (Figure 1), and driver mutations were detected in 39 (25.5%) patients, including EGFR mutations, ALK translocations, and ROS1 rearrangements in 29 (18.9%), 7 (4.9%), and 3 (2.0%) patients, respectively. Of the 39 patients with driver mutation‐positive LA‐NSCLC, 13 (33.3%) received TKI therapy besides definitive CRT before disease progression. The baseline disease characteristics are summarized in Table 1. Patients without driver mutations (wild‐type group) were more likely to be male, smokers, and diagnosed with squamous NSCLC. Otherwise, the baseline characteristics were generally balanced between patients with driver mutation‐positive receiving additional TKI therapy (TKI group) or not (non‐TKI group).

**TABLE 1:** Patient characteristics

	Driver mutation‐positive (*n* = 39) %		Wild‐type group (*n* = 114) %	*p*
	TKI group (*n* = 13)	Non‐TKI group (*n* = 26)		
Age (years)				
Median (range)	62 (46–69)	59 (43–79)	63 (36–86)	0.461
Sex				
Male	5 (38.5)	11 (42.3)	98 (86.0)	<0.001
Female	8 (61.5)	15 (57.7)	16 (14.0)	
ECOG PS				
0	4 (30.8)	11 (42.3)	3 (2.6)	0.570
1	9 (69.2)	15 (57.7)	111 (97.4)	
Stage				
IIIA	3 (23.0)	8 (30.8)	56 (49.1)	<0.001
IIIB	6 (46.2)	10 (38.5)	45 (39.5)	
IIIC	4 (30.8)	8 (30.8)	13 (11.4)	
Histology				
SCC	1 (7.7)	0 (0)	31 (27.2)	<0.001
Non‐SCC	12 (92.3)	26 (100)	83 (72.8)	
Smoking				
Yes	0 (0)	4 (15.4)	54 (47.4)	0.001
No	13 (100)	22 (84.6)	60 (52.6)	
PET–CT				
Yes	6 (46.2)	18 (69.2)	62 (54.4)	0.437
No	7 (53.8)	8 (30.8)	52 (45.6)	

Abbreviations: ECOG PS, Eastern Cooperative Oncology Group performance status; SCC, squamous cell carcinoma; TKI, tyrosine kinase inhibitor.

### 3.2 Survival analysis: impact of driver mutation status and TKI therapy

After a median follow‐up of 26.0 (1–75) months, 95 patients developed initial disease progression, and 72 died. The median PFS and OS of the whole population were 11.0 (95% confidence interval [CI]: 9.0–13.0) and 40.0 (95% CI: 32.1–47.9) months, respectively. The 1‐, 3‐, and 5‐year PFS and OS rates of the whole population were 83%, 62.1%, and 24.8% and 87.6%, 61.4%, and 41.8%, respectively.

**FIGURE 2:**
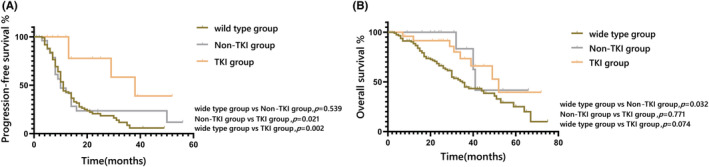
Progression‐free survival (A) rates and overall survival (B) rates in groups stratified by the oncogene mutation

Compared with patients without driver mutations, those in the non‐TKI group had a similar mPFS (12.0 vs 11.0 months, *p* = 0.539) but with a prolonged mOS (52.0 vs 35.0 months, *p* = 0.032). Additionally, compared with patients in the non‐TKI group, those in the TKI group had a significantly longer mPFS (29.0 vs 12.0 months, *p* = 0.021; Figure 2A) but a similar mOS (*p* = 0.771) (Figure 2B). The correlation of common clinicopathological parameters with mPFS and mOS in the whole population are presented in Tables S1 and S2, respectively.

### 3.3 Pattern of treatment failure

**FIGURE 3:**
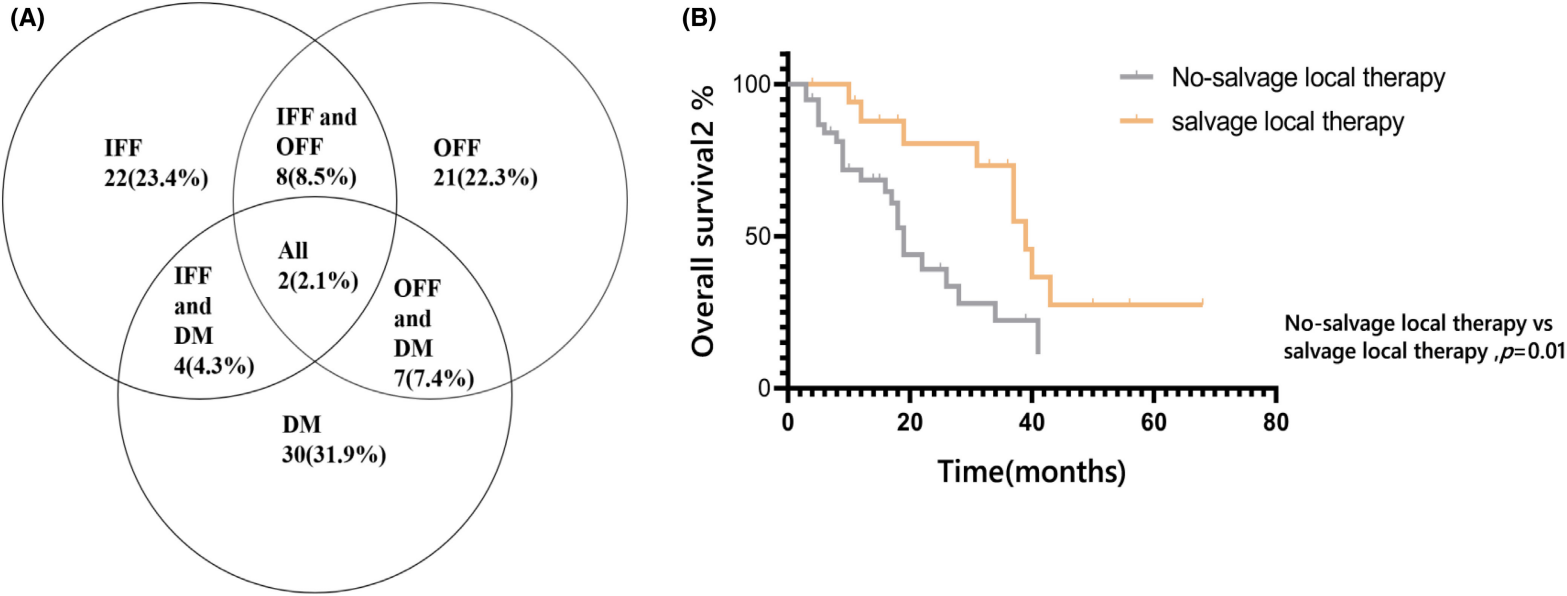
Patterns of initial treatment failure (A) and overall survival (B) rates in patients with oligo‐recurrent disease. DM, distant metastasis; IFF, in‐field failure; OFF, out‐of‐field failure.

By the time of data cut‐off, 95 patients developed their initial disease progression, and the patterns of initial treatment failure are summarized in Figure 3A. In‐field failure alone, out‐of‐field failure alone, and distant metastasis alone were documented in 22 (23.4%), 21(22.3%), and 30 (31.9%) patients, respectively. Simultaneous in‐field and out‐field failure, simultaneous in‐field failure and distant metastasis, and simultaneous out‐field failure and distant metastasis developed in 8 (8.5%), 4 (4.3%), and 7(7.4%) patients, respectively. Two (2.1%) patients concurrently developed in‐field failure, out‐of‐field failure, and distant metastasis. Notably, regarding the frequency of these three types of disease progression, no significant difference was found between the three groups (wild‐type, TKI, and non‐TKI groups).

### 3.4 Clinical value of salvage local therapy in patients with ORD

A total of 57 patients had ORD, including 16 (28.1%) who had isolated BMs and 41 (71.9%) who had limited extra‐cranial recurrence, including 21 (36.8%) and 38 (66.7%) who developed in‐field and out‐of‐field failure, respectively. No significant difference in the percentage of patients developing ORD in the three groups was noted. Of the 57 patients with ORD, 17 (29.8%) received salvage local therapy, including cranial radiotherapy and extra‐cranial local interventions in 8 (14.0%) and 9 (15.8%) patients, respectively. Compared with patients harboring ORD although did not receive salvage local therapy (*n* = 40), those receiving salvage local therapy had significantly longer OS2 (39.0 vs 19.0 months, *p* = 0.01; Figure 3B). Salvage local therapy remained a significant prognostic factor for improved OS2 after adjusting for the potential confounding factors (Table 2).

**TABLE 2:** Univariate and multivariate analyses of OS2

Variables	Univariate analysis	*p*	Multivariate analysis	*p*
	HR (95% CI)		HR (95% CI)	
Sex (female vs male)	1.472 (0.820–2.643)	0.195		
Age (≤60 vs >60)	1.607 (0.975–2.649)	0.063	0.989 (0.961–1.017)	0.427
ECOG PS (0 vs 1)	1.814 (0.442–7.445)	0.408		
Smoking (never vs ever)	1.069 (0.670–1.706)	0.779		
Stage (IIIA vs IIIB vs IIIC)	1.319 (0.966–1.800)	0.081	1.469 (0.973–2.217)	0.067
Histology (non‐SCC vs SCC)	1.103 (0.620–1.961)	0.739		
Driver mutation (− vs +)	0.527 (0.277–1.004)	0.052	0.463 (0.236–0.909)	0.025
Baseline PET–CT (− vs +)	0.846 (0.530–1.349)	0.482		
Salvage LT (− vs +)	0.411 (0.185–0.916)	0.030	0.321 (0.166–0.621)	0.001

Abbreviations: CI, confidential interval; HR, hazard ratio; ECOG PS, Eastern Cooperative Oncology Group performance status; LT, local therapy; OS2, post‐progression survival; SCC, squamous cell carcinoma.

## 4 DISCUSSION

The impacts of driver mutation status on the survival outcomes and patterns of disease recurrence among patients with unresectable LA‐NSCLC and receiving definitive CRT remain controversial. In this study, we found no significant difference in PFS and recurrence patterns among patients with or without driver mutations when receiving only definitive CRT. However, those with driver mutations had a prolonged OS (52.0 vs 35.0 months, *p* = 0.032), most probably owing to salvage TKI use. Meanwhile, in patients with driver mutations, TKI administration before disease recurrence could prolong PFS, although not OS, when compared with definitive CRT alone. Moreover, the majority of recurrent diseases presented as isolated BMs or limited extra‐cranial recurrence, which could derive extra survival benefits from proper local interventions. Taken together, our study provided meaningful data to help optimize treatment modalities for patients with LA‐NSCLC, particularly regarding the clinical value of TKI therapy before disease recurrence for those with driver mutations and salvage local therapy for those with oligo‐recurrence.

The impacts of driver mutation status on survival outcomes and recurrence patterns remain under debate. Patients with EGFR mutations who were treated using CRT alone were found to have similar response, RFS, and short‐term RFS rates compared with those without EGFR mutations in two retrospective studies.^11^, ^29^ However, in other studies, patients with EGFR mutations were revealed to have significantly longer^30^ or shorter PFS.^10^ In all of these four previous studies, patients with EGFR mutation were associated with better local control and higher frequency of BM. A meta‐analysis, including seven studies and 695 patients, demonstrated that for patients with LA‐NSCLC treated with definitive CRT, no significant difference in the ORR between patients with or without EGFR mutations was noted; however, patients with EGFR mutation had a significantly lower rate of local recurrence and a higher rate of BM.^31^ Regarding patients with ALK mutation treated with definitive CRT alone, one previous study found similar recurrence patterns between the ALK‐positive and ALK‐negative patients.^29^ Regarding OS, patients with driver mutations generally had longer OS, particularly after the administration of corresponding TKI therapy.^11^, ^29^, ^30^, ^32^ In the current study, patients with driver mutations had generally similar ORR, RFS, and treatment failure patterns to those without driver mutations when treated with definitive CRT alone. To further clarify these issues, future studies with larger sample sizes are warranted.

TKI therapy administered before disease recurrence could generally prolong RFS; however, its impact on OS remained controversial for patients with driver mutation‐positive LA‐NSCLC and treated with definitive CRT. Single‐agent gefitinib therapy followed by CRT demonstrated acceptable toxicities and favorable efficacy with a 2‐year OS rate of 90.0% and 1‐ and 2‐year PFS rates of 58.1% and 36.9%, respectively.^33^ In a randomized prospective study, patients using erlotinib with concurrent thoracic radiotherapy had significantly longer PFS than those receiving standard CRT among patients with EGFR‐mutant LA‐NSCLC.^25^ Moreover, in a retrospective multi‐center study of 435 patients with EGFR‐mutant LA‐NSCLC, those who received definitive thoracic radiotherapy and EGFR–TKI with or without chemotherapy were associated with improved PFS (21.6 vs 12.7 months, *p* < 0.001) and a tendency of prolonged OS (hazard ratio = 0.65, *p* = 0.072) compared with those who received definitive CRT alone.^34^ In our study, patients receiving a certain kind of additional TKI therapy (most patients received consolidative TKI therapy) had longer PFS than those receiving only definitive CRT, which was generally consistent with the results of previous studies when the treatment sequence was not considered. However, it failed to improve OS, most possibly owing to the commonly used salvage TKI therapy in metastatic disease settings. A phase 3 trial comparing definitive CRT followed by maintenance osimertinib with placebo is ongoing (LAURA, NCT03521154), and the results are highly anticipated.

The disease state of oligometastases is common in advanced NSCLC, and accumulating evidence support the administration of local ablative therapies.^35^, ^36^, ^37^, ^38^ In patients with NSCLC and brain‐only metastasis, significant long‐term PFS and OS could be obtained by aggressively treating primary lung tumors and metastatic sites using surgery or stereotactic radiotherapy.^39^ In patients with NSCLC with limited extra‐pulmonary metastases, including adrenal gland metastasis, relatively long OS and local control rates were obtained after treatment with SBRT.^40^ In our study, recurrent disease that developed as isolated BMs or limited extra‐cranial tumor spreading were jointly termed as oligo‐recurrence as BMs could be treated with surgery or cranial radiotherapy and limited extra‐cranial tumor lesions could be covered using SBRT. We found that 57 (60%) of the 95 patients with disease progression had oligo‐recurrence, and salvage local therapy increased OS2, supporting a potential role of salvage local interventions among patients with LA‐NSCLC, which needs to be further confirmed in larger clinical trials.

This study had several limitations. First, as durvalumab consolidation could significantly prolong PFS in patients without driver mutations, those receiving durvalumab consolidation were excluded to make the comparisons of survival outcomes among patients with or without driver mutations much more straightforward; however, this remains controversial among those with driver mutations.^3^, ^4^ However, the recurrence patterns of patients receiving durvalumab consolidation were generally unchanged compared with those receiving only definitive CRT,^41^, ^42^ indicating that salvage local therapies may still be of great significance even after durvalumab consolidation and are warranted to be further explored. Second, the sample size of patients with driver mutations was limited, and the conclusions needed to be interpreted with caution, particularly for those with ALK and ROS1 translocations.

## 5 CONCLUSION

In patients with inoperable LA‐NSCLC and treated with definitive CRT, the status of driver mutations generally does not impact PFS and the recurrence patterns. TKI therapy beyond definite CRT could improve PFS, although not OS, among patients with driver mutations. The majority of patients with initial disease recurrence would present with isolated BMs or limited extra‐cranial tumor recurrence (jointly termed as oligo‐recurrence), of whom salvage local therapy may provide an additional survival benefit.

## AUTHOR CONTRIBUTIONS

**Jinmeng Zhang:** Conceptualization (equal); data curation (equal); formal analysis (equal); funding acquisition (equal); investigation (equal); methodology (equal); project administration (equal); resources (equal); software (equal); supervision (equal); validation (equal); visualization (equal); writing – original draft (equal); writing – review and editing (equal). **Jiuang Mao:** Conceptualization (equal); data curation (equal); formal analysis (equal); methodology (equal). **Dayu Xu:** Data curation (lead); formal analysis (equal); methodology (equal). **Shanshan Jiang:** Data curation (equal); formal analysis (lead); methodology (equal). **Tiantian Guo:** Data curation (equal); formal analysis (equal); methodology (lead). **Yue Zhou:** Data curation (equal); funding acquisition (equal); methodology (equal). **Li Chu:** Data curation (equal); formal analysis (equal); methodology (supporting). **Xi Yang:** Data curation (equal); formal analysis (supporting); methodology (equal). **Xiao Chu:** Data curation (supporting); formal analysis (equal); methodology (equal). **Jianjiao Ni:** Conceptualization (equal); data curation (equal); formal analysis (equal); investigation (equal); methodology (equal); project administration (equal); resources (equal); supervision (equal). **Zhengfei Zhu:** Conceptualization (equal); data curation (equal); formal analysis (equal); investigation (equal); methodology (equal); project administration (equal); resources (equal); software (equal); supervision (equal); validation (equal); visualization (equal).

## CONFLICT OF INTEREST

There is no conflict of interest regarding the publication of this article.

## ETHICAL STATEMENT

The authors are accountable for all aspects of the work in ensuring that questions related to the accuracy or integrity of any part of the work are appropriately investigated and resolved. Our study followed The Declaration of Helsinki (as revised in 2013). The institutional review board of Fudan University Shanghai Cancer Center approved the study (approval number 090977‐1). Informed consent was waived by the Institutional Review Board because this was a retrospective study.

## Supporting information


Table S1.

Table S2.

## Data Availability

The datasets used and/or analyzed during the current study are available from the corresponding author on reasonable request.
