# Supplementary material for: Pattern of failure and clinical value of local therapy for oligo‐recurrence in locally advanced non‐small cell lung cancer after definitive chemoradiation: Impact of driver mutation status
Source: Cancer Med. 2022 Dec 16;12(6):6971–9. doi: 10.1002/cam4.5493 (PMC10067091; doi:10.1002/cam4.5493)
Supplement: Supplementary file 1 — Table S1. [file CAM4-12-6971-s001.docx]

| **Supplemental Table 1 Univariate and multivariate analysis of PFS** | | |
| --- | --- | --- |
| Variables | Univariate analysis |  |
|  | HR (95% CI) | *P* |
| Sex (female *vs.* male) | 1.314(0.818-2.112) | 0.258 |
| Age (≤ 60 *vs*. ＞60) | 1.190(0.793-1.787) | 0.401 |
| ECOG PS (0 *vs.*1) | 0.742(0.103-5.331) | 0.767 |
| Smoking (never *vs.* ever) | 1.175(0.792-1.745) | 0.423 |
| Stage (IIIA *vs.* IIIB *vs.* IIIC) | 0.831(0.630-1.096) | 0.190 |
| Histology (Non-SCC vs SCC) | 1.285(0.781-2.114) | 0.324 |
| Driver mutation (-*vs.*+) | 0.580(0.357-0.942) | 0.028 |
| Baseline PET-CT (-*vs.*+) | 0.907(0.610-1.349) | 0.631 |

PFS, progression-free survival; HR, hazard ratio; CI, confidential interval; ECOG PS, Eastern Cooperative Oncology Group performance status; SCC, squamous cell carcinoma.
