# Supplementary material for: Pattern of failure and clinical value of local therapy for oligo‐recurrence in locally advanced non‐small cell lung cancer after definitive chemoradiation: Impact of driver mutation status
Source: Cancer Med. 2022 Dec 16;12(6):6971–9. doi: 10.1002/cam4.5493 (PMC10067091; doi:10.1002/cam4.5493)
Supplement: Supplementary file 2 — Table S2. [file CAM4-12-6971-s002.docx]

**Supplemental Table 2 Univariate and multivariate analysis of OS**

|  | Univariate analysis |  | Multivariate analysis |  |
| --- | --- | --- | --- | --- |
|  | HR (95% CI) | *P* | HR (95% CI) | *P* |
| Sex (female *vs.* male) | 1.552(0.865-2.785) | 0.141 |  |  |
| Age (≤60 vs. >60) | 1.573(0.957-2.585) | 0.074 | 1.459 (0.886-2.403) | 0.137 |
| ECOG PS (0 vs.1) | 2.163(0.527-8.886) | 0.284 |  |  |
| Smoking (never vs. ever) | 1.078(0.675-1.719) | 0.754 |  |  |
| Stage (IIIA vs. IIIB vs. IIIC) | 1.090(0.801-1.485) | 0.583 |  |  |
| Histology (Non-SCC vs SCC) | 1.477(0.825-2.643) | 0.189 |  |  |
| Driver mutation (-vs.+) | 0.424(0.223-0.806) | 0.009 | 0.444(0.233-0.848) | 0.014 |
| Baseline PET-CT (-vs.+) | 0.779(0.489-1.244) | 0.296 |  |  |

OS, overall survival; HR, hazard ratio; CI, confidential interval; ECOG PS, Eastern Cooperative Oncology Group performance status; SCC, squamous cell carcinoma.
